# Partial Water Intrusion and Extrusion in Hydrophobic Nanopores for Thermomechanical Energy Dissipation

**DOI:** 10.1021/acs.jpcc.4c02900

**Published:** 2024-07-11

**Authors:** Gonçalo Paulo, Luis Bartolomé, Oleksandr Bondarchuk, Simone Meloni, Yaroslav Grosu, Alberto Giacomello

**Affiliations:** † Dipartimento di Ingegneria Meccanica e Aerospaziale, Sapienza Università di Roma, 00184 Rome, Italy; ‡ Centre for Cooperative Research on Alternative Energies (CIC energiGUNE), Basque Research and Technology Alliance (BRTA), 01510 Álava, Spain; ¶ International Iberian Nanotechnology Laboratory, 4715-330 Braga, Portugal; § SPIN-LAB Centre for microscopic research on matter, University of Silesia in Katowice, 75 Pułku Piechoty 1A St., bldg J, 41-500 Chorzów, Poland; ∥ Dipartimento di Scienze chimiche, farmaceutiche ed agrarie, Università degli Studi di Ferrara, 44121 Ferrara, Italy; ⊥ Institute of Chemistry, University of Silesia in Katowice, Szkolna 9, 40-006 Katowice, Poland

## Abstract

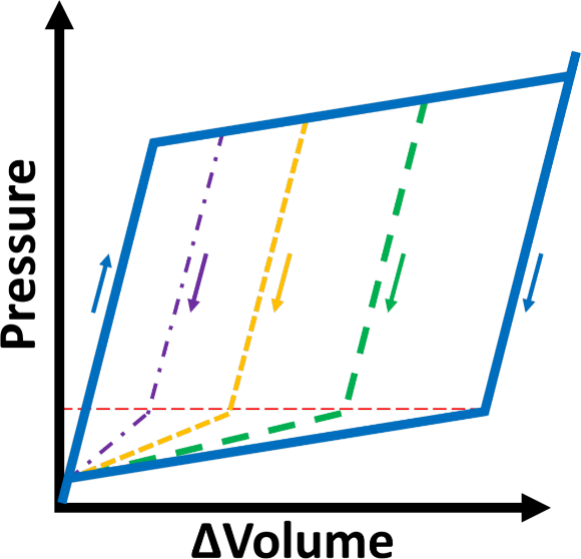

Forced wetting (intrusion) and spontaneous dewetting (extrusion) of hydrophobic/lyophobic nanoporous materials by water/nonwetting liquid are of great importance for a broad span of technological and natural systems such as shock-absorbers, molecular springs, separation, chromatography, ion channels, nanofluidics, and many more. In most of these cases, the process of intrusion-extrusion is not complete due to the stochastic nature of external stimuli under realistic operational conditions. However, understanding of these partial processes is limited, as most of the works are focused on an idealized complete intrusion-extrusion cycle. In this work, we show an experimental system operating under partial intrusion/extrusion conditions and present a simple model that captures its main features. We rationalize these operational conditions in terms of the pore entrance and cavity size distributions of the material, which control the range of intrusion/extrusion pressures.

## Introduction

Intrusion and extrusion in heterogeneous lyophobic systems (HLS), i.e., the pressure-induced process of a liquid penetrating a lyophobic porous matrix and the opposite process of the liquid emptying the pores at lower pressures, is important for multiple technological and industrial applications.^1,2^ The intrusion–extrusion cycle generally exhibits hysteresis: a difference between the intrusion pressure at which the nonwetting liquid enters the nanopores upon compression and the extrusion pressure at which the nonwetting liquid leaves the nanopores upon decompression.^3^

The difference between intrusion and extrusion pressures determines the technological applicability of HLS. For example, when the extrusion pressure is significantly lower than the intrusion pressure, the intrusion–extrusion process can be used to dissipate energy because the mechanical energy of compression is higher than the released energy upon decompression. Thus, a typical application of HLS displaying this behavior is for shock absorption;^4−8^ to this end, different porous materials were used with water or aqueous solutions such as mesoporous grafted silicas^9−12^ or metal–organic frameworks (MOFs).^13^ Moreover, as an alternative to the aqueous solutions and water, glycerin and glycerol,^14,15^ ferromagnetic fluids,^16^ and ionic liquids^17^ were used for HLS-based shock absorber technologies.

However, if intrusion and extrusion pressures are almost identical, i.e., the pressure hysteresis is low, the behavior of the HLS would be similar to a spring, being capable of storing energy. This nonhysteretic behavior is promising to develop molecular spring technologies for energy storage,^18,19^ liquid pistons,^20^ and thermal actuation.^21^ Regrettably, only very few porous materials exhibit this nonhysteretic behavior with water or aqueous solutions: mainly zeolites,^22−24^ but also some MOFs.^25^ Therefore, in order to advance HLS applications, it is crucial to develop new porous materials with high stability or strategies for tuning the intrusion–extrusion hysteresis.^26−29^

Besides mechanical energy, the intrusion–extrusion cycle implies comparable exchanges of thermal energy.^30−32^ This heat generation is potentially useful for thermal energy storage applications.^33^ Furthermore, apart from compression–decompression cycles, the intrusion–extrusion in HLS can be achieved by heating–cooling cycles.^34^ This characteristic becomes suitable for developing thermal actuation applications or technologies.^21,35^ Finally, intrusion–extrusion in HLS is also relevant for several other applications such as (i) chromatography,^29,36−100^ (ii) negative compressibility,^39−101^ (iii) triboelectric generators,^42,43^ and (iv) biological systems.^44,45^

**Figure 1:**
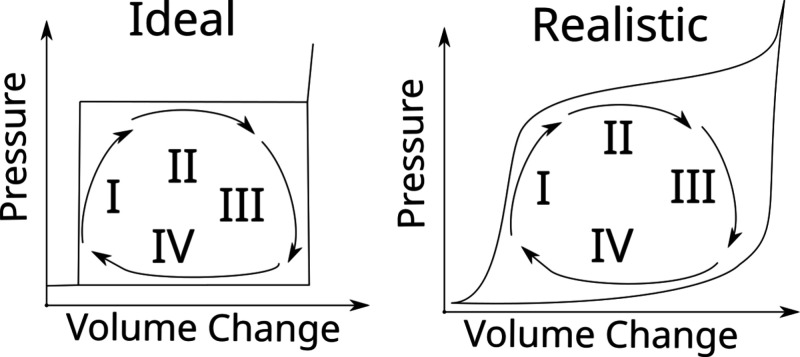
Ideal and realistic representation of intrusion/extrusion isotherms. A liquid porosimetry cycle is characterized by a pre-intrusion branch (I), an intrusion branch (II), a pre-extrusion branch (III) and an extrusion branch (IV). In the ideal representation of this process, the system is incompressible, represented by the branches I and III being vertical, and the intrusion and extrusion pressures have a single defined value, represented by the branches II and IV being horizontal. In a more realistic representation of this experiment, the system is partially compressible, and branches I and III have a slight curvature, and there is a distribution of intrusion and extrusion pressures, seen in the slope of branches II and IV.

In real-life applications of intrusion–extrusion, HLS are expected to work over a broad range of frequencies and pressures. Therefore, the porous matrix of the HLS must be chosen such that intrusion and extrusion occur within the operating pressure ranges. Partial intrusion(extrusion) occurs when compression is stopped before total intrusion is achieved (or decompression is stopped before total extrusion). This happens because, in realistic situations, there is not a single intrusion or extrusion pressure, but a range of them, see Figure 1. For this reason, it is central for a very broad range of applications to characterize the behavior of HLS under partial intrusion/extrusion and to identify the material properties that control it.

In this work, we show experimental realizations of an HLS {water + C8-grafted silica}, which undergoes partial intrusion and extrusion. In hydrophobic pores of suitable size and chemistry, as in this work, depending on the pressure value, the intruded and extruded states are separated by a free-energy barrier, which may be significantly higher than the system’s thermal energy. Thus, intrusion occurs when the pressure is high enough to overcome the barrier, rather than when the wet state is more stable than the dry one.^46^ An analogous argument works for extrusion in the opposite direction: extrusion may occur at a pressure lower than the thermodynamic coexistence. These strong metastabilities are at the origin of the intrusion/extrusion hysteresis. Taking into account this behavior, we construct a simple theoretical model quantifying the intrusion and extrusion barriers of hydrophobic nanopores and use it to generate dynamic simulations, which follow an intrusion/extrusion protocol similar to the experiments. This model reproduces the qualitative behavior of partial intrusion and partial extrusion, with the advantage of being able to link it to material properties. In particular, we observe that a distribution of pore entrance radii, which results in distribution of intrusion pressures, is necessary for the partial intrusion to occur. Moreover, we also find that a second, independent material parameter needs to be considered to entirely explain the shape of the intrusion and extrusion cycle.

## Methods

### Experiments

#### Materials

The HLS used in this paper consists of water and a commercial nanoporous silica grafted with a hydrophobic coating used for column chromatography known as SymmetryPrep C8, supplied by WATERS (referred to as WC8 in the text). The grafting was done with octylsilanes with a density of 2.1 groups/nm^2^ according to the data provided by the supplier. Distilled water was used for intrusion–extrusion experiments.

#### Porosimetry Experiments

H_2_O intrusion–extrusion tests were carried out employing a water porosimeter. The WC8 silica mixed with water was encapsulated into a flexible hermetic polymeric capsule before the testing. This capsule was subjected to compression–decompression cycles using Auto Pore IV 9500 porosimeter (Micromeritics Instrument Corporation, Norcross, USA). The penetrometer was evacuated to a pressure less than 7 Pa, followed by filling with mercury to the corresponding pressures. The silica was subjected to partial water intrusion-extrusion cycles done at the same rate, but, for partial intrusion, the maximum applied pressure was progressively increased from 16 to 30 MPa in six steps, and, for partial extrusion, the minimum applied pressure was reduced in seven discrete steps from 2 to 0.1 MPa. For reference, 30 and 0.1 MPa are the pressures where total intrusion and extrusion are reached, respectively. These experiments were also used to characterize the pore entrance distribution of WC8. For this purpose, the water intrusion branch recorded in the 0.1–30 MPa pressure range was converted into pore entrance distribution using the classical approach via Laplace law, known surface tension of water and contact angle value of 120°. For these experimental tests, as usual, the very first cycle was ignored due to minor irreversibilities upon the first intrusion, such as the removal of some physiosorbed grafting and leftover residues from synthesis, formation of defects both in grafting and in silica and minor quantities of water that stay in metastable state. All those factors account for 1–2% of change in the intrusion volume in the first cycle, but for a clean comparison, it still makes sense to ignore it. Then, three cycles were performed, which were highly repeatable. Two experimental campaigns were conducted, i.e., two separate loadings of fresh material into the cell. The results of these two campaigns are similar, with only minor differences in the overall compressibility of the hydraulic systems, which contain slightly different quantities of water in the different loadings. Before the intrusion-extrusion experiments, different calibrations were performed, including a blank experiment compressing water to account for its compressibility. The compressibility of silica was tested by comparing its compressibility in the preintrusion region, as it might be different from the compressibility of filled silica (grafted or nongrafted). In the pressure range reported in this work, the compressibility of both water and silica can be considered independent of pressure.

#### Characterization of WC8

**Figure 2:**
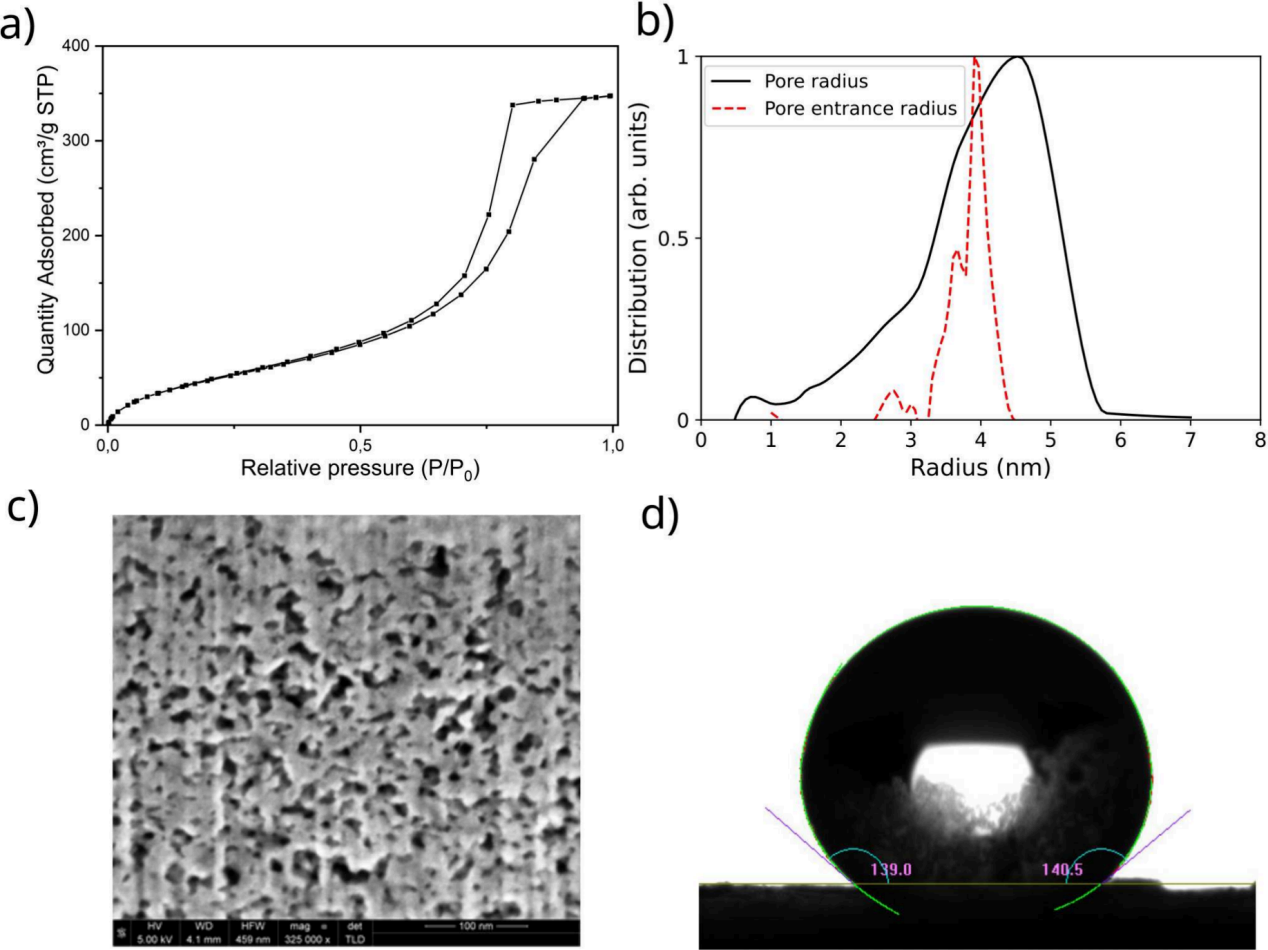
Characterization of WC8. Panel a) shows the results of Nitrogen sorption experiments in WC8 samples. Panel b) describes the pore radius distribution obtained using the Barrett–Joyner–Halenda model and the pore entrance radius distribution obtained using the Laplace equation in independent water intrusion experiments. Panel c) represents a cross-sectional view of WC8 particles using SEM. The nanoporous structure is characterized by spherical pores interconnected randomly. Panel d) shows contact angle measurements of water wetting of silica WC8.

Textural properties were characterized in an automated gas adsorption analyzer (Micromeritics ASAP 2460). Nitrogen sorption curves of the samples were measured under isothermal conditions after outgassing at 200 °C in vacuum for 5 h, Figure 2a. The multipoint surface area was evaluated with the Brunauer–Emmett–Teller method over the range *P*/*P*_0_ = 0.075–0.35, where *P*_0_ is the saturated pressure of nitrogen, and the pore radius distribution was obtained using the Barrett–Joyner–Halenda model applied to the desorption isotherm branch, Figure 2b. The total pore volume was determined from the volume adsorbed at *P*/*P*_0_ = 0.98.

FEI Helios NanoLab 450S DualBeam - Focused ion beam (FIB) with FEG SEM was used to record micrographs of WC8. For this purpose, the FIB technique was deployed to observe the cross-sectional view of WC8 particles, Figure 2 c). First, Pt protective layer  was deposited above a WC8 particle via electron beam (3 keV) stimulated decomposition of a Pt-containing gaseous precursor. Next, proximity half of the particle was sputtered away using a focused Ga ion beam (30 keV, 9.6 nA). Finally, polishing of the cross-section surface was performed by low-current (30 keV, 100 pA) focused Ga ion beam followed by 52° tilt of the sample for the cross-section analysis.

To determine the contact angle for water wetting of silica WC8 using the sessile drop technique, Figure 2d, the material (60 mg) was pelleted pressing at 1 ton for 30 min using a die of 13 mm. The contact angle measurements were conducted by LAUDA Surface Analyzer LSA 100 (LAUDA Scientific GmbH) using the software SurfaceMeter.

### Theory

#### Free Energy of Intrusion and Extrusion

**Figure 3:**
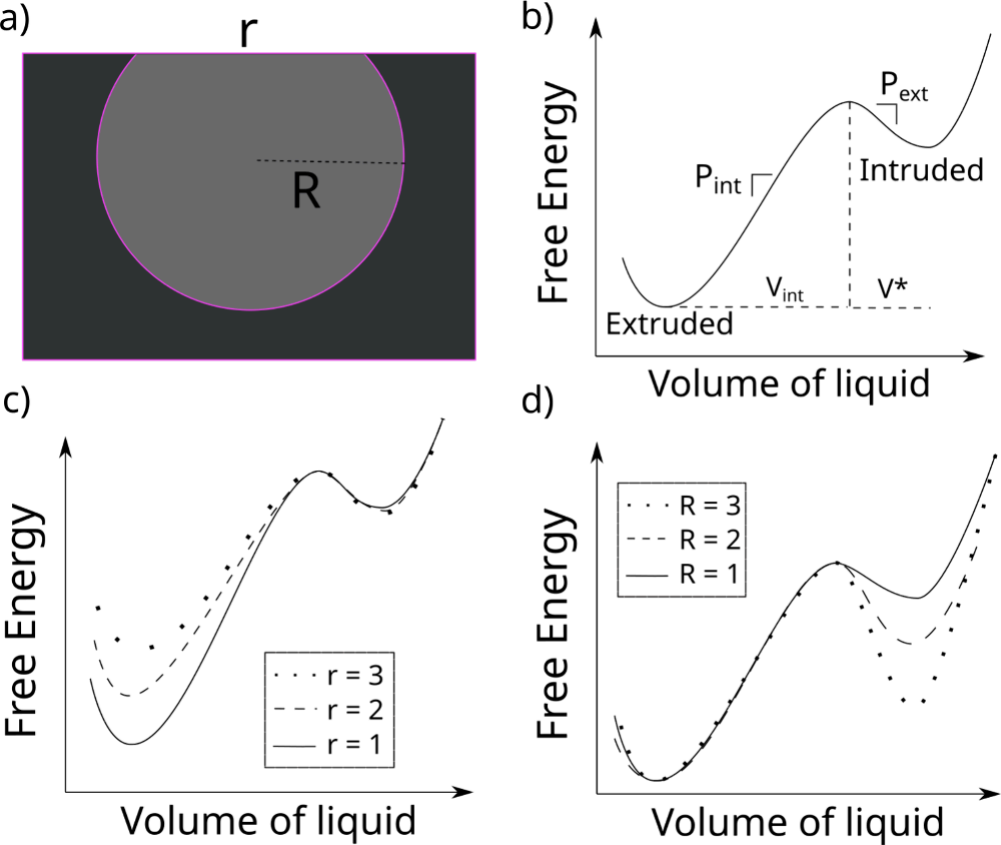
Description of the model of the free energy of intrusion and extrusion. Panel a) shows a schematic of the spherical pore, with pore radius *R*, and pore entrance radius *r*. The pore is characterized by a contact angle *θ*. The free-energy profile for such type of pores is represented in panel b). The intrusion pressure corresponds to the slope at the inflection point in the profile that connects the bottom of the free energy, the extruded state, to the maximum.^46^ The extrusion pressure also corresponds to the absolute value of the slope at the inflection point in the free energy profile, but in the branch that connects the minimum corresponding to the intruded state to the free energy maximum. These inflection points define the maximum and minimum “tilting” Δ*PV* by which the reference free energy profile can be changed before the extruded and intruded minima disappear, respectively.^46^ Panel c) shows that the intrusion pressure increases as the pore entrance radius is decreased, because the slope of the left part of the free energy profile is increasing. Panel d) shows that the extrusion pressure decreases as the radius is increased.

The pore topology of WC8 is well described by randomly intersecting spheres, see Figure 2c. Consistent with this, and as done in other works,^38^ we will consider that it is possible to describe the intrusion and extrusion dynamics by modeling the pores of the materials as spherical cap pores. The main factors determining the intrusion and extrusion dynamics are (i) the radius of the pore, which determines the size of the pore, (ii) the radius of the base of the cap, which determines the radius of the pore entrance, and (iii) the hydrophobic capacity of the material, see Figure 3.

For sufficiently large pores, the pressure required for intrusion of a liquid into a hydrophobic porous material, *P*_int_, is given by the Young–Laplace equation:
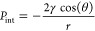
1where *γ* is the liquid–vapor surface tension of the intruding liquid, *θ* is the Young contact angle of the liquid as measured in a flat slab of the same solid constituting the porous matrix, and *r* is the radius of the pore entrance. This means that the intrusion pressure is sensitive only to the size (*r*) and chemistry (*θ*) of the pore entrance radius, but not to the more distant portion of the pore, see Figure 3c.

No simple expression relating the extrusion pressure to the chemical and geometrical characteristics of the material, similar to the Young–Laplace equation, exists despite the fact that extrusion experiments have been performed using different materials, with different contact angles and pore entrance radii. Evidently, the extrusion pressure is (equal or) smaller than the intrusion pressure and, for cylindrical pores, decreases as the pore radius increases up to a point where no extrusion occurs above atmospheric pressure.^3^ In this work, we will assume that the extrusion pressure depends on a single factor: the radius of the spherical pore, *R*, which is consistent with previous results showing that the curvature of the pore is a determining factor for the nucleation of a vapor bubble^47^ in cavities.

The presence of subnanometric apertures in the pore wall may change the effective contact angle of the pore,^38^ and the amount of these cavities may depend on the pore radius. This more subtle phenomenon is not taken into account in the model. However, this effect would still lead to a dependence of the extrusion pressure on the pore radius. The microscopic origin of this relation goes beyond the objective of this work and is not discussed in the following.

The extrusion process is characterized by the formation of a bubble of a critical volume, V*, after which the expansion of said bubble is energetically favored. In this work, we modeled V* as being one-quarter of the total volume of the spherical pore (before removing the cap), such that the critical volume depends only on the pore radius. Other fractions could be used, but the precise value does not change the overall picture. Summarizing, the above analysis suggests that all considered microscopic phenomenona imply a dependence of *P*_ext_ from the pore radius *R*, instead of the pore entrance radius *r*, see Figure 3d.

With these ingredients, we construct a simple model representing the free-energy profile corresponding to the intrusion and extrusion processes. Because we focus on the variation of the number of water molecules inside the pores, which we link to the intruded volume, we use the grand potential, Ω(μ, *V*, *T*), as our free-energy functional. The intrusion pressure depends only on the intrusion barrier, Ω_int_, and on the critical intrusion volume, *V*_crit_, which is the volume of the pore minus the critical volume for extrusion, *V*_int_ = *V* – V*. By construction, this means that the intrusion pressure is the one at which the intrusion barrier disappears. Accordingly, the extrusion pressure is also the one at which the extrusion barrier Ω_ext_ disappears.

We illustrate, in the Supporting Information, Figure S2, the free-energy profiles used for different pore entrance radii and sphere radii. We also show that the expected characteristics of a monodisperse material, i.e., one that has a single pore entrance radius, Figure S3.

#### Langevin Dynamics

Having a model for the free energy profile associated with the intrusion and extrusion processes for a given pair of pores radius and pore entrance radius, we are now interested in simulating the evolution of the intrusion and extrusion processes replicating the experimental protocol. Because intrusion and extrusion are thermally activated processes, the transition is a stochastic event, i.e., it occurs randomly, when an energy fluctuation allows overcoming the barrier separating the intruded and extruded states. Thus, we decide to model them using stochastic dynamics. As done in the previous section for the free energy, we have used the volume of liquid inside the pore, *V*, as the variable to describe the state of the system–the progress of the intrusion/extrusion process. We will consider that it is possible to describe the dynamics of *V* using an overdamped Langevin equation:^48^
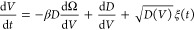
2where ξ(*t*) is a white noise process, *D*(*V*) is the diffusivity associated with this process and Ω is the free energy, which is being used as the potential of mean force. Previous work has shown that for simple model nanopores, the diffusivity, *D*, is not constant in the filling variable.^48^ Although modeling *D* is necessary if one is interested in quantitatively reproducing effects related to time, this is not necessary for the present work, and we chose to use *D* = 3*nm*^6^/*ns*, a value of the same order of magnitude as what has been found for cylindrical nanopores.^48^

To model the effect of pressure on the free energy of a state corresponding to a certain volume, we consider that Ω(*V*) increases by an amount equal to Δ*PV*,^46,49,50^ where Δ*P* is the external pressure applied to the system. We remark that the “tilting” term Δ*PV* is added to the reference free-energy profile (at Δ*P* = 0) such that, at each value of *V*, the interfaces are those minimizing surface free energy and can therefore be considered fixed. This simple law allows taking into account the pressure dependence of the thermodynamic forces driving intrusion/extrusion. Having access to a model of the free-energy profile describing the intrusion process, and to a model of how pressure affects it, we simulated the intrusion and extrusion processes by computing the free energy profile at a certain applied pressure, and integrating the Langevin equation using the Euler-Mayorama^51^ algorithm, using a time step of 0.1 ns. The pressure was increased or decreased, according to protocols that mimic the experimental ones.

## Results

### Partial Intrusion

**Figure 4:**
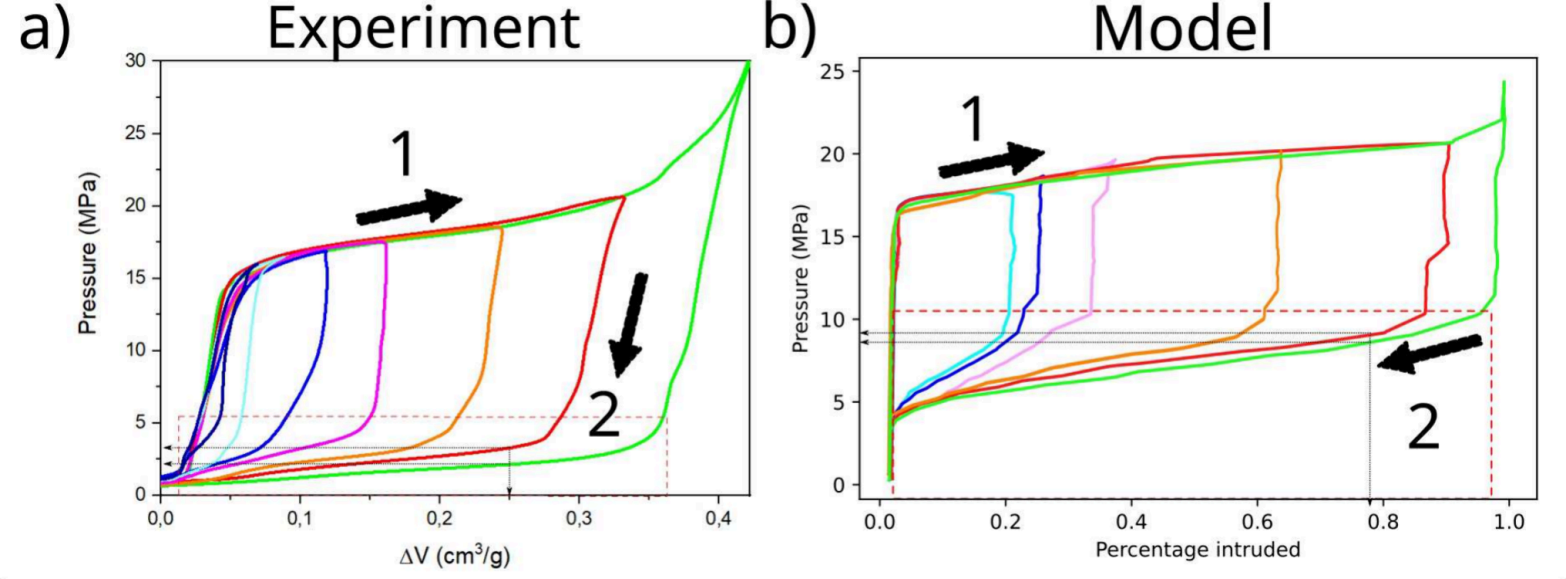
Partial intrusion in {WC8+water} system and model prediction. a) Pressure cycles performed at the same rate, but with a progressive increase of the maximum applied pressure *P*_max_ (from 16 MPa in dark blue to 30 MPa in green). As higher pressures are applied, the material is further intruded (larger volume change), showing that intermediate filling levels are possible. b) We performed Langevin simulations using a pressure protocol similar to the experimental one. To compute the free-energy profiles used in the Langevin simulations, we considered a pore entrance radius distribution similar to the experimental distribution, and we considered that the spherical pores have a Gaussian size distribution. In both plots, arrows represent the direction of the cycle.

Partial intrusion was observed in intrusion/extrusion cycles on a commercial silica gel grafted with a hydrophobic coating – WC8– see Figure 4a. This material has been extensively characterized before,^38^ and is known to have a broad distribution of pore radii. Scanning electron microscopy reveals that WC8 has a disordered topology, and the pores are characterized by randomly intersecting spheres, see Figure 2c.

The intrusion-extrusion cycles were performed by quasistatically changing the system volume while logging the increasing/decreasing pressure. To study partial intrusion, the pressure was increased, at a rate of 1 MPa/s, until reaching a certain target pressure *P*_max_ higher than *P*_int_, the pressure of onset of intrusion, i.e., when the intrusion (almost horizontal) plateau starts. Then, pressure was decreased at the same rate of the intrusion step down to atmospheric pressure. Partial intrusion, i.e., the wetting of only a fraction of the WC8 cavities, was observed (Figure 4a) with increasing intruded fractions for growing values of *P*_max_. This is due to the Young–Laplace law, eq 1, which prescribes that only cavities with suitable pore entrance radii can be wet, *r* < – 2 cos *θ*/*P*_max_. Since the pore entrance radius is polydisperse, only a fraction of the pores is intruded.

The system is subjected to several values of maximum pressures *P*_max_. Regardless of *P*_max_, immediately after starting the decompression step no extrusion is observed, i.e., the initial reduction of pressure is accompanied by a limited change of volume, which is only due to the compressibility of porous material and water. When the system reaches a threshold pressure, it extrudes, exhibiting large volume variations. This pressure, denoted *P*_ext_, is empirically determined by the intersection of the lines fitting the vertical and horizontal domains of the extrusion branch. Remarkably, *P*_ext_ is independent of the maximum pressure reached in the intrusion step *P*_max_. For very low values of pressure with *P*_max_ close to *P*_int_, the intrusion/extrusion cycle is irregular and one cannot draw reliable conclusions. An important observation is that the extrusion branches corresponding to different *P*_max_ do not overlap. In other words, Figure 4a shows that, at a given pressure lower than *P*_ext_, but before extrusion is completed, the volume of the heterogeneous system depends on *P*_max_, i.e., pressure and temperature are not sufficient to characterize the state of the system, as usually assumed in thermodynamics, but they depend on the experimental history.

To highlight the exceptional nature of this phenomenology, let us perform a *thought experiment*. Consider bulk water vapor and slowly increase pressure above the saturation pressure, *P*_sat_. Vapor condenses. Next, start reducing pressure. When the system crosses again the saturation pressure, liquid water evaporates. Regardless of the maximum pressure reached during the compression, in the decompression step, evaporation starts at the same pressure, *P*_sat_. According to thermodynamics, a pair of pressure and temperature values define a single state of the system. One can think of the intrusion and extrusion branches of liquid porosimetry as confined condensation and evaporation, respectively.^52^Figure 4a shows that *confined* condensation and evaporation of water within WC8 is qualitatively different from the usual thermodynamic notion: although evaporation always starts at the same value, the volume of the system does not depend only on the current temperature and pressure but is also strongly history dependent, as quantified by the maximum pressure *P*_max_ reached in the intrusion cycle (see Figure 4a, gray arrows). Below, our model provides an explanation of the apparent violation of the thermodynamic behavior by HLS.

As previously assumed for WC8,^38^ our porous material is modeled as independent pores with a spherical cap geometry, which are defined by two (almost) independent parameters: the pore entrance radius *r* and the radius of the sphere *R*. Our model simulates independently each combination of these two parameters, see Methods, and we average the intrusion and extrusion results over a pore radius and pore entrance radius distribution to mimic the experimental conditions. For the pore entrance radius distribution, we use a Gaussian distribution with an average value of 3.9 nm and a standard deviation of 0.2 nm, similar to the experimental distribution obtained via water porosimetry (Figure 2b). As discussed in detail in the *Theory* section, to model extrusion, one needs also the distribution of pore sizes. We assume that the pore radii are distributed according to a Gaussian with an average value and standard deviation compatible with the corresponding values of the pore entrance radius discussed above, i.e., with *r* < *R*. In particular, *R* is sampled from a Gaussian distribution with an average value of 4.6 nm and a standard deviation of 0.6 nm according to pore size distribution obtained via gas adsorption, see Figure 2b.

Figure 4b shows the simulated partial intrusion procedure to be compared with the experimental one in Figure 4a. The results of the model are in qualitative agreement with experimental results. Samples partially intruded at different values of *P*_max_ extrude always at the same value, *P*_ext_, but extrusion branches of the cycle in the *P*-Δ*V* domain do not overlap. In particular, the higher *P*_max_, the lower the pressure required to achieve the same Δ*V* (Figure 4b, gray arrows) upon decompression. Our model provides the following explanation of the *P*_max_-dependence of the extrusion process. The distribution of *r* and *R*, *p*(*r*, *R*), is correlated, i.e., it is not simply the product of the (overall) pore mouth and pore sizes. However, there is no one-to-one correspondence between *r* and *R*. Intrusion is a phenomenon occurring at the pore entrance of the pores and thus depends on *r*. Extrusion instead starts in the interior of the cavity and is thus defined by the pore size *R*. Large pores may be associated with small entrances, which are intruded only at high *P*_max_. As we discussed above, larger pores require lower pressures to be extruded. Thus, in the extrusion branch, to achieve the same Δ*V*, which requires extruding these large pores with narrow entrance, one needs to reach lower pressures (Figure 4b, gray lines). In figure S1 of the Supporting Information, we show that the main peaks of the derivative of the volume with respect to pressure are at similar pressures for all *P*_max_.

We note that the accord of experimental and simulation results in Figure 4 is not quantitative. Indeed, 1) the intrusion and extrusion pressures were not tuned to reproduce the experimental values and 2) the pre-intrusion and pre-extrusion branches of the simulated cycle are steeper than the experimental ones because we do not include the compressibility of the HLS system, which is of course intrinsic in experiments, although we attempted to compensate for the compressibility of the system (including the porosimeter) from the experimental curves. With considerable simulation effort, one could finely tune the characteristics of the model to quantitatively match the experimental results for a specific system, but the objective of this work is rather explaining the physical origin of the partial intrusion/extrusion cycles and the apparent violation of the thermodynamic behavior.

### Partial Extrusion

**Figure 5:**
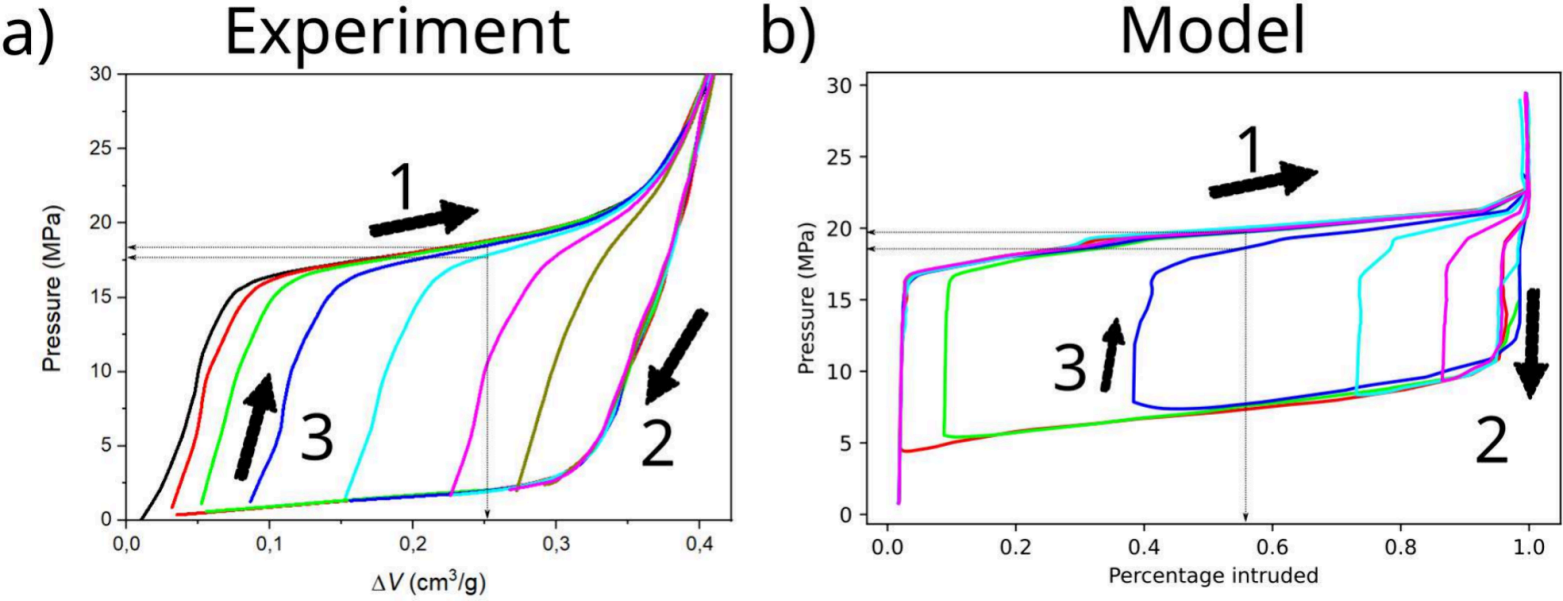
Partial extrusion in {WC8+water} system and model prediction. After completely wetting the material (black line, labeled with “1”), the pressure is reduced to progressively lower values (from 2 MPa in brown to 0.1 MPa in red, labeled with “2”), before raising it again to 30 MPa (labeled with “3”). The system progressively extrudes as the pressure is lowered, again showing that intermediate filling levels are possible. If pressure is risen before full extrusion, the intrusion process starts over again. b) We perform Langevin simulations using a pressure protocol similar to the experimental one. To compute the free energy profiles used in the Langevin simulations, we consider the same pore entrance radius distribution and pore radius distribution as in Figure 4. In both plots, arrows represent the direction of the intrusion/extrusion cycle.

Experiments were also performed to pinpoint the origin of partial extrusion in hydrophobic nanoporous materials. Again, the material was the grafted silica gel WC8, but, in this case, the experimental protocol started with the completely intruded material, i.e., at an applied pressure of 30 MPa. The pressure was reduced down to the target pressure *P*_min_ at a rate of 1 MPa/s; subsequently, the pressure was increased again to 30 MPa at the same rate. The cycle was then repeated using different values of *P*_min_, see Figure 5a. Similar to partial intrusion, when the extrusion process was incomplete, intrusion did not occur immediately after starting compression but only when a threshold pressure was reached, empirically determined by the intersect of the lines fitting the vertical and horizontal domains of the intrusion branch. This threshold was found to be independent of *P*_min_ and to coincide with *P*_int_. Analogous to partial intrusion, in partial extrusion, the intrusion branches corresponding to different values of *P*_min_ do not coincide (see Figure 5a, gray lines): the lower *P*_min_, the higher the pressure one has to apply to achieve the same Δ*V*.

Using the same modeling approach described in the previous section, we performed simulations of the experimental protocol to capture the observed partial extrusion behavior. In particular, we used the same pore entrance and pore radius distribution used in the partial intrusion model discussed in the previous section. We recall that the pore entrance radius *r* determines the intrusion pressure of a pore, while the radius *R* of the pore itself controls the extrusion one. Simulations qualitatively reproduce the partial extrusion phenomenology observed experimentally (compare panels a and b of Figure 5). When the pressure is increased after a partial extrusion, intrusion starts at the same critical pressure *P*_int_ as for the complete intrusion process, independently of *P*_min_. Despite the independence of the intrusion pressure on *P*_min_, analogous to the partial intrusion case, in partial extrusion, the intrusion branches of the cycle do not overlap for simulations carried out at different values of *P*_min_. The explanation is analogous to the one given for partial intrusion and depends on the distributions of pore characteristics–the distribution of pore entrance radii of the nonintruded pores as a function of *P*_min_. Indeed, in a cycle defined by lower values of *P*_min_, more pores are extruded, namely larger pores, and some of them have narrow pore entrances. To (re)intrude the pores with narrow entrance radii one needs higher pressures, which explains why, for a given Δ*V*, the plateau in the intrusion branch are shifted to higher pressures for lower *P*_min_.

### Partial Intrusion and Extrusion in Different Systems

**Figure 6:**
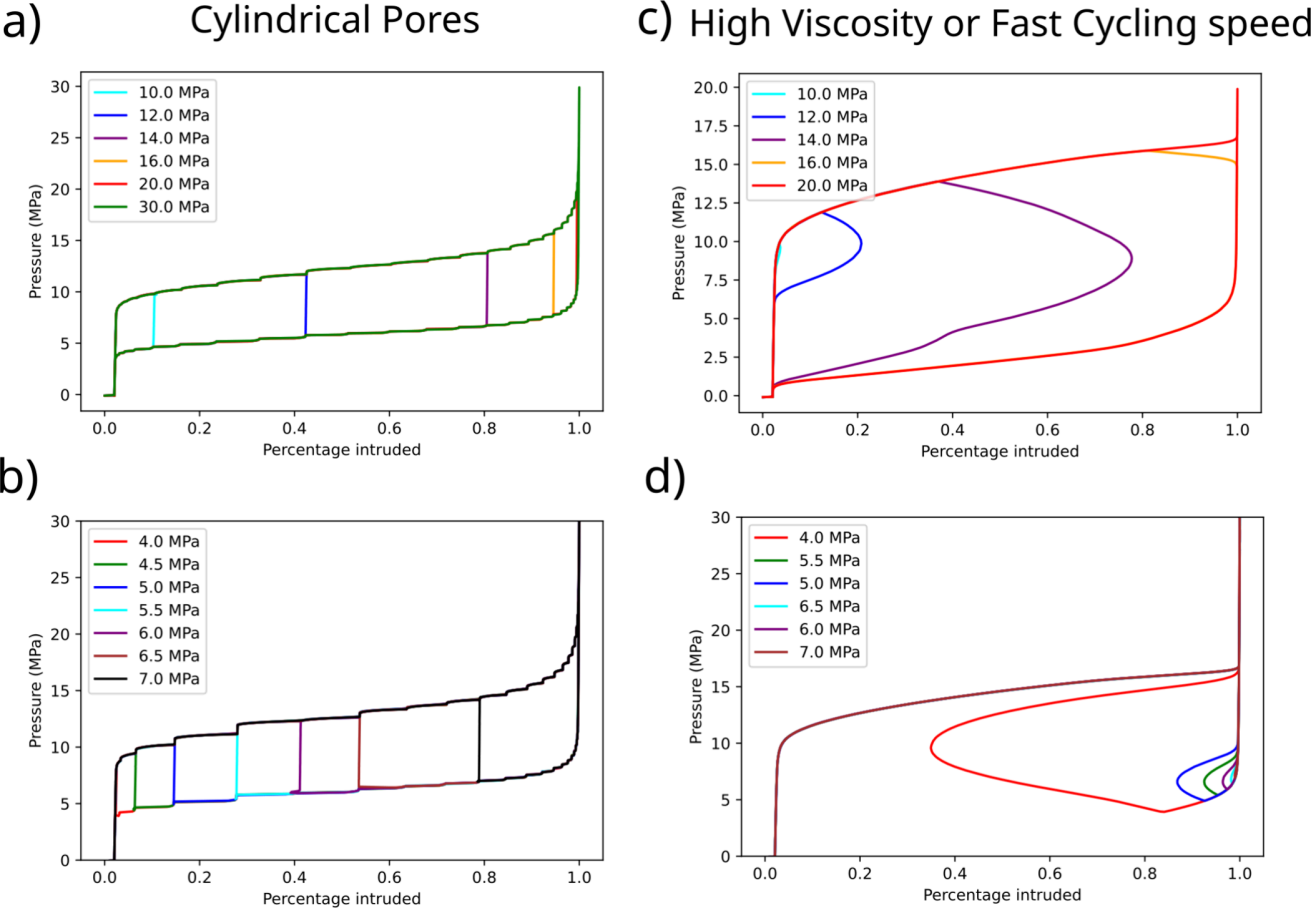
Partial intrusion and extrusion in different systems. Panel a) also shows the same partial intrusion simulation protocol as in Figure 4 but considering that intrusion and extrusion pressures depend both on the same parameter, in this case, the pore entrance radius. Panel b) shows partial extrusion simulations considering intrusion and extrusion pressures depend only on the pore entrance radius. Panel c) shows that partial intrusion can be observed even for systems with no pore entrance radius distribution if the cycling experiment is done at a frequency comparable with the intrusion time. Panel d) shows the equivalent simulation but for partial extrusion.

The model developed above allows us also to predict other possible origins of partial intrusion and extrusion by hypothesizing that the intrusion and extrusion pressures depend on different parameters, not seen in WC8. For instance, in the case of cylindrical nanopores, like MCM-41,^3^ both the intrusion and extrusion pressures should depend on a single physical parameter, the pore radius. We explore this effect in Figure 6a and b, showing that this scenario leads to a qualitatively different partial extrusion behavior. In both partial intrusion and extrusion simulations, the slopes of the intrusion (extrusion) branches are independent of *P*_max_ (*P*_min_), contrary to what is observed for the WC8 case. This can be easily rationalized using the arguments given above: large pores with narrow pore entrances cannot exist for MCM-41 due to its cylindrical geometry; therefore, a single parameter (the pore radius) defines both the intrusion and extrusion pressures.

Partial intrusion and extrusion may have an even more general origin. Because both intrusion and extrusion are thermally activated, stochastic events^1^ that are favored by increasing the pressure, even an ideally monodisperse material, e.g., cylinders having a single radius, do not exhibit all-or-none intrusion at the pressure predicted by the deterministic Kelvin-Laplace eq 1 as shown in Figure 1a. The same applies for extrusion. In other words, there is a time distribution of intrusion and extrusion events, which results in a partial intrusion or extrusion for sufficiently fast experiments. This phenomenon is normally not observed experimentally because intrusion and extrusion barriers are generally large as compared to the thermal energy and vary quite rapidly with the pressure, resulting in a narrow range of pressures in which these stochastic processes are possible. Despite this, one can expect that, when the intrusion and extrusion times are comparable to the cycling time, e.g., if the intrusion-extrusion cycles are very fast or if high viscosity liquids^17^ are used, it may be possible to observe partial intrusion and extrusion, as shown in Figure 6c and d.

## Conclusions

Understanding the conditions at which partial intrusion and extrusion are observed is fundamental in applications to guarantee the correct functioning of HLS under realistic operating conditions, which include a variable range of pressures. We demonstrate that partial intrusion and extrusion occur when there is a distribution of intrusion and extrusion pressures. Moreover, we show that, at a variance with thermodynamic intuition, the state of an HLS is not fully characterized by two thermodynamic variables, say temperature and pressure. Rather, the state of a HLS is strongly history-dependent, i.e., it varies with the maximum and minimum pressures achieved during the intrusion and extrusion processes, respectively.

For the specific nanoporous materials considered in this work, WC8, the distribution of intrusion pressures originates in the intrinsic pore entrance radius distribution, but there are other possible factors that could create a distribution of intrusion pressures, like the size of crystallites in metal–organic frameworks.^28^ We hypothesize that, in WC8, due to the shape of the pores, the extrusion pressure is mostly affected by the size of the pores rather than by their entrance size. Materials with cylindrical nanopores, like MCM-41, can also show partial intrusion and extrusion, but we show with our model that the shape of the intrusion and extrusion cycles are expected to be qualitatively different. We show that even truly monodisperse pores can show partial intrusion or extrusion if the cycling speed of the experiment is close to the expected time for the intrusion or extrusion processes.

Summarizing, a combination of experimental and theoretical results have clarified (i) under what conditions it is possible to stop an intrusion–extrusion cycle in HLS at intermediate volumes and restart it subsequently; (ii) that partial intrusion/extrusion results in a history-dependent behavior different from condensation/evaporation of bulk equilibrium systems; (iii) the general features of the partial intrusion/extrusion behaviors are found to depend on the geometrical parameters of the nanoporous material and on extrinsic parameters such as the cycling time, which could be useful to engineer real-world energy applications of HLS.

## Supporting Information Available

A more detailed analysis of the extrusion pressure distribution in partial intrusion experiments and details about the free energy profiles used in our models (PDF)
