# Supplementary material for: Partial Water Intrusion and Extrusion in Hydrophobic Nanopores for Thermomechanical Energy Dissipation
Source: J Phys Chem C Nanomater Interfaces. 2024 Jul 11;128(29):12036–45. doi: 10.1021/acs.jpcc.4c02900 (PMC11284848; doi:10.1021/acs.jpcc.4c02900)
Supplement: Supplementary file 1 — jp4c02900_si_001.pdf [file jp4c02900_si_001.pdf]

Supporting information - Partial Water Intrusion and Extrusion in Hydrophobic Nanopores for Thermomechanical Energy Dissipation

Gonçalo Paulo,[†,#] Luis Bartolomé,[‡,#] Oleksandr Bondarchuk,[¶,§] Simone Meloni,[*,‖] Yaroslav Grosu,[*,‡,⊥] and Alberto Giacomello[*,†]

†Dipartimento di Ingegneria Meccanica e Aerospaziale, Sapienza Università di Roma, 00184 Rome, Italy

‡Centre for Cooperative Research on Alternative Energies (CIC energiGUNE), Basque Research and Technology Alliance (BRTA), 01510 Álava, Spain

¶International Iberian Nanotechnology Laboratory,4715-330 Braga, Portugal

§SPIN-LAB Centre for microscopic research on matter, University of Silesia in Katowice, 75 Pułku Piechoty 1A St., bldg J, 41-500 Chorzów, Poland

‖Dipartimento di Scienze chimiche, farmaceutiche ed agrarie, Università degli Studi di Ferrara, 44121 Ferrara, Italy

⊥Institute of Chemistry, University of Silesia in Katowice, Szkolna 9, 40-006 Katowice, Poland.

#These authors contributed equaly

E-mail: simone.meloni@unife.it; ygrosu@cicenergigune.com; alberto.giacomello@uniroma1.it

Extrusion pressure distribution for partial intrusion experiments

Figure S1 shows the derivative of the volume to pressure to show clearly when the extrusion occurs for the partial intrusion tests. The main peak of extrusion occurs around 2.5 MPa for all the cases - colors here correspond to the ones in Figure 4a) of the main text.

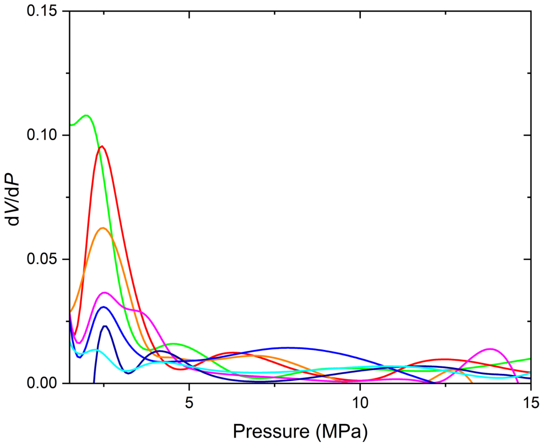

Fig. S1: Extrusion pressure distribution for partial intrusion experiments. Colors of each curve correspond to the ones in Figure 4a) of the main text.

Free energy profiles for the simulation model extrusion

We construct a simple model representing the free energy profile corresponding to the intrusion and extrusion processes. In figure S2 a) and b) we show some representative free energy profiles used to simulate intrusion and extrusion cycles for systems with different pore entrance radius and pore radius. As explained in the main text, the intrusion and extrusion pressures can be extracted from the slopes of the profiles and their dependences on the pore entrance radius and on the pore radius are shown in panels c) and d). Increasing the pore entrance radius makes intrusion easier, as intrusion pressure becomes lower, and increasing the pore entrance radius makes extrusion harder, as extrusion pressure also goes down.

As mentioned in the main text, when the system is mono disperse, having always the same pore

radius and pore entrance radius, partial intrusion and extrusion are not possible, see figure S3.

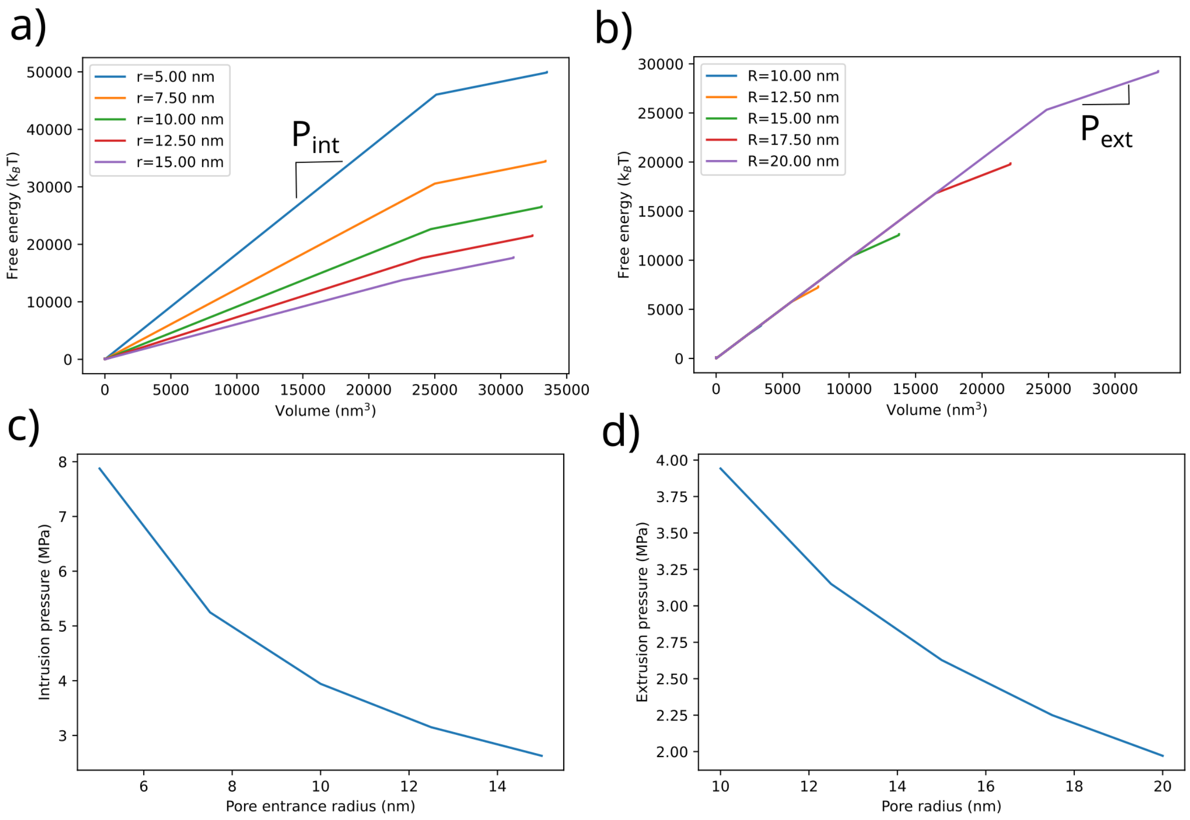

Fig. S2: Free energy profiles at different pore radius and pore entrance radius. Panel a) shows the dependence of the free energy profile on the pore entrance radius, r, for different values of intruded volume. The slope of the right part of the profile gives the intrusion pressure, P_int, which is represented in panel c). Panel b) shows the dependence of the free energy profile on the pore radius, R, for different values of intruded volume. The slope of the left part of the profile gives the intrusion pressure, P_ext, which is represented in panel d).

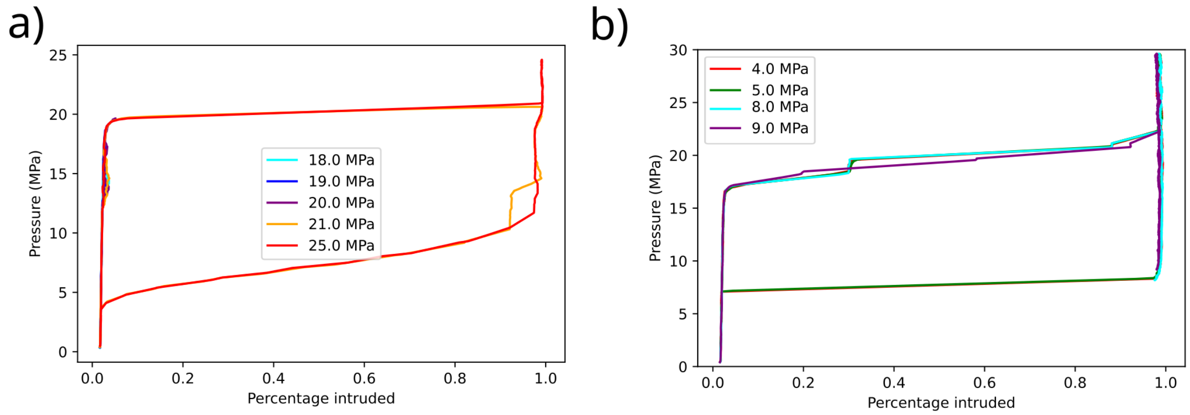

Fig. S3: Mono-disperse systems behave differently. Panel a) shows the same simulation protocol as Figure 4 b) of the main text, considering a system with a single pore entrance radius but the same pore radius distribution. This case leads to no partial intrusion. Panel b) shows the same simulation protocol as Figure 5 b) of the main text, considering a system with a single pore radius but a pore entrance radius distribution. This case leads to no partial extrusion.
